# Moral panic and gaming disorder: focused on the mediation role of negative attitudes toward digital games

**DOI:** 10.3389/fpsyg.2025.1614807

**Published:** 2026-01-02

**Authors:** Sung Je Lee, So Young Kim, Christopher J. Ferguson, Eui Jun Jeong

**Affiliations:** 1 Department of Digital Culture and Contents, Konkuk University, Seoul, Republic of Korea; 2 Global Creative Industry Research and Evaluation Center, Hankuk University of Foreign Studies, Seoul, Republic of Korea; 3 Department of Psychology, Stetson University, DeLand, FL, United States

**Keywords:** moral panic, gaming disorder, socio-cultural concerns, negative attitudes toward youth, negative attitudes toward games

## Abstract

**Introduction:**

With the inclusion of “Gaming Disorder (GD)” in the official disorder manual by the World Health Organization (WHO), the concept of “Moral Panic (MP)”—referring to preemptive regulation driven by the spread of socio-cultural concerns—has attracted significant attention in related debates. However, empirical evidence regarding the MP phenomenon remains scarce. This study examined how socio-cultural concerns (responsibility to media threats and negative attitudes toward youth) and negative attitudes toward games are associated with the degree of socio-cultural agreement on the policy of pathologizing GD.

**Methods:**

A structural equation model was tested using survey data from 2,000 participants recruited in South Korea.

**Results:**

Negative attitudes toward youth and responsibility to media threats were positively associated with negative attitudes toward games and support for regulation. Notably, negative attitudes toward games mediated the relationships between these socio-cultural concerns and agreement with pathologizing GD. In addition, being female, older, and spending less time gaming were associated with more negative attitudes toward games.

**Discussion:**

These findings suggest that socio-cultural concerns play a critical role in shaping public support for regulatory policies by fostering negative attitudes toward new media. They also highlight the importance of considering MP-related processes when interpreting public responses to GD.

## 1 Introduction

The World Health Organization (WHO) included pathological gaming, specifically excessive gaming that disrupts users’ everyday social lives, into the 11th version of International Classification of Diseases (ICD-11) with the name of “Gaming Disorder (GD).” After the WHO’s decision, the academic community has been actively debating the degree to which this disorder reflects both research data and clinical utility (e.g., Aarseth et al., 2017; Brand et al., 2019). This debate is accelerating further as the spread of digital devices has increased and the universalization of digital-based interactions has become a reality since the epidemic of COVID-19 (Ferguson, 2020). Indeed, some studies have reported that many people chose digital games to alleviate social isolation and negative emotions during the COVID-19 period (Wang et al., 2023), and gaming disorder is mentioned as one of the negative outcomes emerging from the process of coping with pandemic-related psychological distress (Guo et al., 2024; Han et al., 2022). The WHO and its supporters assert that GD has spread to a level of socio-cultural concern, particularly among adolescents, necessitating professional intervention (e.g., Brand et al., 2019). Above all, the WHO proposes that digital game usage can directly cause pathological harm, especially to adolescent users, and therefore, special measures should be taken under the name of “gaming disorder.” However, existing research on gaming disorder has primarily emphasized its pathological characteristics, whereas studies addressing broader socio-cultural dynamics remain limited (Chew et al., 2024). This not only hinders the development of practical interventions and systems for gaming disorder, which is ambiguously positioned between game regulation and health policies, but it can also make it difficult to achieve a social consensus that transcends cultural or generational differences in perspective.

Accordingly, some scholars have expressed concern about the decision since excessive anxiety and preemptive regulations of games can also suppress the potential of games in terms of leisure, education, and culture, and can hinder the drawing of socio-cultural consensus to properly utilize digital games (e.g., Aarseth et al., 2017). They are also concerned that game discourse could be limited due to “Moral Panic (MP)” which refers to precautionary regulation due to societal fears not based on empirical verification. Additionally, MP could also cause harm to gamers both through stigmatization, but also through the promulgation of useless treatments that fail to identify underling issues that cause excessive gaming such as depression or ADHD (Quandt, 2017). This perspective highlights the need to examine gaming disorder as a socio-cultural discourse. In other words, an approach from a socio-cultural discourse perspective not only influences the understanding of and development of social consensus on gaming disorder, but it can also assist in establishing health policies and industry-related systems. However, research to date has rarely attempted to illuminate moral panic through empirical methods or to examine whether related factors can actually influence the establishment of regulatory policies.

According to previous studies, MP can be developed with the spread of such socio-cultural concerns over media threats and regarding youth mainly by older generations who are unfamiliar with new media and technology. This then leads to negative attitudes toward the designated new media (e.g., digital games), and finally results in the issue of regulation policies toward the media (Przybylski, 2014). Moral panic has long been associated not only with the various cultural phenomena of the younger generation, who are labeled as deviant rule-breakers, but also with the society-wide overreaction of forming negative discourse and moral regulation against them (Critcher, 2008). In other words, MP not only stigmatizes gamers—who have failed to prove their value in established society and neoliberal cultural spaces or are culturally marginalized—as “disposable youth,” but it also shapes social discourse to agree on “disposing” of digital games, their primary form of leisure culture, as pathological or unproductive (Chen et al., 2025; Giroux, 2012). Such regulation policies due to socio-cultural concerns or fear persist in some countries such as China and South Korea, and the intensity of regulation is gradually being strengthened (Feiner and Kharpal, 2021).

However, now that gaming disorder is recognized as a disease, digital game culture and the game industry are in a situation where they must respond to new regulatory policies or the spread of negative perceptions—all while protecting their leisure and industrial value—even before a social consensus is reached. Especially in a situation that demands discussions based on objective results and deliberation to avoid undermining the intrinsic value of digital games or to minimize harm, a lack of understanding of moral panic could lead to excessive regulation or unnecessary damage. Therefore, it is necessary to understand the main factors that cause media moral panic, its formation process, and the process by which it leads to regulation, and to investigate this empirically. Against the policies, gaming industry associations have argued and asked to remove the policies which regulate or oppress the gaming industry. For example, in Korea, laws had been instituted such that games could not be used after midnight for adolescents under 15 due to the fear of gaming addiction. Following these controversies, the legal ban known as the “Forced Shutdown System,” which enforced restrictions on youth gaming in South Korea, was repealed in 2022. However, evidence ultimately emerged that these regulatory effectives were not helpful for adolescent sleep or mental health (Lee et al., 2017). Despite ongoing debates, the empirical evidence on the effects of socio-cultural concerns as development logics of MP is rather scarce.

Based on the concept of MP, this paper was designed to empirically investigate the relationships among socio-cultural concerns (i.e., responsibility to media threats and negative attitudes toward youth), negative attitudes toward digital games, and the degree of socio-cultural agreement with the policy of pathologizing GD by examining a structural equation model with a sample of 2,000 subjects from South Korea. In the model, we also tested whether negative attitudes toward digital games mediate between the socio-cultural concern variables and agreement to pathologizing gaming disorder.

## 2 Literature review

### 2.1 Moral panic phenomena

Moral Panic (MP) is a concept that explains excessive reactions to socio-cultural problems (Rohloff and Wright, 2010). It implies that such concerns and threats about a specific target could be distorted and keep on spreading. In the process of promoting psychological fear of negatively perceived events or characters, objective or factual examination of the object could be excluded. According to Cohen (2002), when an object is regarded as a threat to the values or interests of a society, the danger of the object is formalized by the media, and “moral barricade” is installed under the name of protecting society. Even in MP, “selective bias” occurs that information supporting such fear and concern is rewarded while the opposite information is suppressed (Gauntlett, 2005; Ferguson, 2010).

Such distorted information affects major social groups, including politicians, journalists, and scholars, and it is continuously circulated in the public as professional policies, reports, and research (Gauntlett, 2005). Ferguson (2008) presented the process and vicious cycle of MP as “moral panic wheel model” in which the MP relates to the substructure of politics, society, culture, and education. Thus, MP, through the information circulation in the wheel, results in solidifying a negative attitude toward a specific target and further in inducing policy change or social practice in society. In addition, since the fear about a specific object spreads rapidly, socio-cultural concerns do not easily disappear even when the controversy surrounding harmfulness is resolved (Goode and Ben-Yehuda, 2009). This implies that MP could act as a stumbling block to the correct understanding of the object and produce long-term effects on society.

New media contributes to the formation of MP since information distortion is rapidly spreading by the new media. Particularly, SNS often function as mediums for MP because, while they allow the public to participate easily, distorted information is readily shared, and the credibility of dispersed information is difficult to verify (Obadă and Dabija, 2022). In addition, the information distortion can be exaggerated in the old generation with the fear of the threat toward minors or youths. As confirmed by Ferguson’s wheel model, the older generation, who function as the audience for the MP, (re)produces and circulates exaggerated information in conjunction with the media. Manipulated information causes a chain reaction that produces social alienation of the target, and new media spreads such information to the public more quickly. On the other hand, unlike the older generation, those who embrace it encompass all classes of society including the youth. Those who are accustomed to new media, thus, can feel another level of panic by a different aspect of the MP. Therefore, it should not be overlooked that the MP surrounding new media can intensify the conflict between generations.

The examples of media and content-based MP include melodrama, movies, jazz, rock & roll, cartoons, animation, television, and digital games as well as Dungeon and Dragon and Harry Potter (Ferguson, 2010; Ferguson and Beaver, 2016; Gauntlett, 2005; Kutner and Olson, 2008). These examples show that the harmfulness of media and content can be disseminated in an exaggerated state much more than the actual damage caused by the target of MP, and can affect socio-cultural awareness and resistance to media use. In this regard, Pascoe (2011) pointed out that the older generation was overly concerned about the online media culture of young people, and argued that fears about new media had been affecting popular culture discussions and policy making since the mid-1990s.

The pattern that the older generation becomes the audience for an MP related to new media results in cultural oppression of the user groups and the industry. Digital games, which are currently at the forefront of new media content, have become a primary hobby for teenagers and younger adults. For the teen players, such games could be a learning place to experience social behaviors in advance. For example, MMORPGs (Massive Multiplayer Online Role-Playing Games) players in their teens can collaborate with other players and achieve their own goals in the games.

Negative attitudes toward youth and responsibility toward media threat to the youth affect concerns and negative attitudes toward digital games, which is the youth’s leisure culture. When games are perceived as a media threat that harms youth, the older generation will actively support legal or social sanctions to protect youth from the media (Przybylski, 2014). Underlying this responsibility is the premise that immature youths may be swayed by harmful media without the help of the older generation. In other words, it is considered that it can lead to the damage of socio-cultural values such as traditional morals as well as individual delinquency.

Thus, the older generation with a strong sense of responsibility toward the youth is more likely to exaggerate the alleged deleterious effects of new media such as digital games and be wary of them. As a result, a ‘moral barrier’ to block threats from youth is formed, which furthermore appears as an institutional regulation on games.

### 2.2 Factors associated to negative attitudes toward games

The first factor associated with negative attitudes toward games is age. The older generation has a much stronger sense of socio-cultural influence and responsibility than the younger generation. This is because older generations have more opportunities to influence social and cultural issues and media interests through voting or newspaper subscriptions than younger generations (Ferguson and Beaver, 2016), and they are more sensitive to child and youth protection issues. In particular, this protective sense of responsibility may lead to overestimating the harmfulness of certain media beyond what empirical evidence supports. Often, this is related to familiarity with the target of the MP more than age per se (Ferguson and Colwell, 2017, 2020). The important point here is that when the older generation’s ignorance of an unfamiliar object is combined with the overprotective consciousness toward the younger generation, it can encourage distorted fear toward the object. Some studies also confirm that the older generation’s sense of responsibility for adolescents is related to their support for media regulation policies. For example, a study conducted on 1,359 parents in the United States revealed that the level of support for social media regulation is more influenced by “moral foundation salience,” such as “care” and “authority,” rather than by political ideology (Vigil et al., 2025). Furthermore, the same study confirmed that in terms of parenting styles, authoritative parenting styles, which require a high level of responsibility and high control, also influence support for media monitoring and regulation policies.

As such, the abstract fear of the older generation toward new media can have a significant impact on negative perceptions of real objects. In particular, the older generation, who fervently worry about the harmfulness of new media and believe that young people should be isolated from it, tend to respond negatively to new media use or demand preemptive policies from relevant institutions and local communities to restrict youth access, even when there is no objective data supporting such fears. In the case of parents who are especially sensitive to issues concerning their children, the positive functions of games may be underestimated, while their children’s media use and the harmful effects of games may be overinterpreted. Related studies have revealed that parents are more likely to perceive games negatively regardless of their children’s actual gaming patterns or psychological functioning and are also more inclined to regard their children as having a gaming disorder. Furthermore, Lieberoth and Fiskaali (2021) point out that explicit parental concerns intended to protect their children may, in fact, exacerbate parent–child conflicts or encourage maladaptive media use, thereby creating the appearance of gaming disorder symptoms that are not necessarily grounded in reality. Due to this, the older generation lacking experience with new media will evaluate the harmfulness of games as more abstractly and excessively dangerous than the actual level of objective concern from reliable data (De Aguilera and Mendiz, 2003) This attitude of the older generation works as a key factor in inducing negative attitudes toward games and can have a significant effect on attitudes toward cultural and political opinions or regulatory policies.

In fact, in a study in New Zealand, it was reported that the parent generation who has not experienced games is more likely to have negative preconceived notions about games, but experience with games can provoke a reevaluation of these concerns (Schott and Van Vught, 2013). These influences are not limited to political and cultural issues and can be similarly applied to academic fields, particularly given many academics, particularly those in positions of authority, are quite old. For example, some researchers and organizations may conduct, or support research aimed at stimulating negative attitudes and fears about digital games, rather than examining objective truth. Overwrought statements of harm are often created by committees of scholars for whom the median age is quite old and not representative of the youth who consume new media (Markey and Ferguson, 2017).

The second issue related to negative attitude toward games is found regarding the gap between the old media generation and the new media generation (Ferguson et al., 2017). Particularly in Western cultures, while adolescents are viewed as subjects requiring protection, they are also considered to be in a period vulnerable to rebellion and irresponsibility. However, the culturally formed stereotype of the ‘irresponsible and immature younger generation’ can lead to unreasonable stigma or excessive control, or even be internalized by the youth themselves, thereby causing self-fulfilling prophecies (Qu, 2023). Moreover, this stereotype formation does not merely remain an individual’s perception of the generation; it can also influence the formation of distorted beliefs among the older generation that young people’s performance or work skills are lacking, and lead to negative portrayals of the younger generation. Furthermore, these stereotypes and negative perceptions of the younger generation influence the undervaluation of their culture, media, and lifestyle, and even lead to controlling and regulatory attitudes toward them (Ferguson, 2015). In particular, a negative attitude toward the younger generation can act as a factor that makes the older generation negatively perceive digital games, the main leisure culture of the ‘game generation’. The negative perception of the younger generation induces them to evaluate their game-playing lifestyle as immature behavior and a problem that needs to be corrected. This lack of understanding toward the game generation can intensify conflicts over media issues by further reinforcing the gap in perception between generations about the harmfulness of games (Beck and Wade, 2004). As such, Ferguson (2015) found that negative attitudes toward digital games are affected not only by age but also by negative attitudes toward the younger generation. Put simply, people who are suspicious of the negative impact of games tend not to like adolescents very much and view them negatively.

As we have seen so far, negative attitudes toward games are largely embodied by two aspects. One is MP formed by excessive fear and protective responsibility toward new media, and the other is the negative perception toward the new media generation. The problem is that distorted perceptions and fears about games tend to be expressed in extreme forms that prevent them from using the game, rather than trying to understand the child’s experience correctly. This acts as a potential cause of conflict between children and parents (Kim and Doh, 2014; Wood, 2008). Therefore, research on games needs to be explored from the perspective of MP that encompasses new media and intergenerational conflict. An objective gaze toward the game and new media generation, this leads to the direction of the content industry that will lead the future society and trust in its protagonists. This is the reason why academic research, which is a major factor in shaping MP, needs to be critically re-examined for the very MP again.

### 2.3 Pathologizing gaming disorder

Despite the existence of negative attitudes toward games due to MP and differences in perceptions between generations, the number of groups enjoying games will increase in the future. Due to COVID-19, new media digital platforms are replacing the daily stage, and it is self-evident that increased online leisure activities will also contribute to the proliferation of games. As the WHO recently decided to classify GD as a type of disorder, gaming and MP have re-emerged. Currently, GD has been officially adopted by ICD-11, and symptoms such as (1) persistent gaming behavior, (2) impaired control over gaming, (3) functional impairment due to gaming have been suggested as key features of lasting more than 12 months (King et al., 2020; Saunders et al., 2017).

On the other hand, there are both pros and cons regarding creating an official diagnosis of GD. First, scholars supporting the morbidity of GD expect that the WHO decision will stimulate new research in key fields such as epidemiology, neurobiology, treatment, prevention, and public health (Billieux et al., 2017; Grajewski and Dragan, 2020). In fact, since the WHO decision, various attempts have been made to elucidate the symptoms and characteristics of GD and the problems that may be caused by GD. According to a recent study, GD is associated with obesity or insomnia (delayed sleep phase syndrome and insomnia), and it is also associated with impulsivity, aggression, and anxiety (Fumero et al., 2020; Ko et al., 2019; Jeong et al., 2019; Rho et al., 2018).

Conversely, some argue that it is premature to recognize GD as a formal mental illness (Aarseth et al., 2017; Przybylski et al., 2017). Such reasons are as follows. First, there is a lack of qualitative and quantitative studies to support the disease development of gaming disorder. This is because the number of clinical studies is insufficient to promote disease development, problems in estimating prevalence or the small sample size (Przybylski, 2016; Van Rooij et al., 2017). Second, the criteria for measuring gaming disorders are vague. In this regard, there are opinions that the concept of GD is overly dependent on substance use and gambling criteria (Aarseth et al., 2017) and the opinion that there is a lack of objective and clear measurement tools. In particular, the proliferation of similar tools and the absence of a unified measurement tool are recognized as problems that must be solved even by scholars who support the disease-making of GD, and in fact, related research is ongoing (Grajewski and Dragan, 2020; King et al., 2020).

Others point out that it is unclear whether GD is triggered by gaming consumption, or because of other disorders or symptoms. For example, some studies have shown that GD is positively correlated with ADHD symptoms (Bae et al., 2016), and other studies have also shown that GD is difficult to assert by the time spent playing games alone (Király et al., 2017). In addition, studies have also found that gaming disorders may be more affected by parental relationships and academic stress than by gaming time (Jeong et al., 2019). This is all to say that, rather than a stand-alone disorder, GD may simply be symptomatic of other, underlying issues, or even an adaptive stress-reducing response to them.

Differences in opinions among scholars on GD are also distinguished from the views on MP. First, the opponents of GD point out that the disease model of GD can stigmatize healthy game users (Király et al., 2017; Ferguson et al., 2017). Specifically, MP regarding digital games can cause a contraction in game culture and negative prejudice toward gamers, and through policy changes, it can shrink the global game community and lead to erroneous behavior in the medical community such as misdiagnosis or application of useless therapies to phantom disorders (Király et al., 2017). According to this, GD’s pathologization based on vague evidence can be abused in a way to instigate MP, and it can have the opposite effect of justifying institutional and cultural oppression and sanctions against games in a distorted way.

Meanwhile, scholars in support of GD point out that GD itself does not treat all gamers as potential patients, so there is no need to be overly wary of the WHO’s decision (Lee et al., 2017). It is also argued that critical attitudes based on MP stem from false beliefs about the harmlessness of games (Lee et al., 2017). Therefore, scholars who are generally favorable to GD give more value to public health benefits than the socio-cultural impact of GD. Above all, the disease of GD underscores the need as a preemptive measure to support those suffering from gaming. The academic confrontation suggests the need for creative research that reflects socio-cultural characteristics for a clear understanding of how people accept or do not accept GD as a diagnostic category.

Meanwhile, a series of studies have indicated that issues related to Gaming Disorder (GD) may stem from intergenerational conflict and control dynamics between parents and children, or more broadly, between older and younger generations. For instance, adults who bear the responsibility of protecting minors—such as parents and teachers—may adopt excessively controlling or monitoring strategies in the course of childrearing or guidance. Such practices can provoke conflicts over media use and, in the long term, exacerbate adolescents’ psychosocial functioning, thereby fostering an overreliance on media. For example, a study involving 452 adolescents reported that higher levels of parental monitoring were associated with increased impulsivity and stress in social situations among adolescents, which in turn led them to use video games as a means of emotional escape (Commodari et al., 2024). Conversely, another study with 595 high school students found that lower levels of parental monitoring were linked to a greater likelihood of exhibiting GD, while factors such as poor family functioning and an anomic perception of the social environment also contributed to the development of GD (Marinaci et al., 2021). Furthermore, a systematic review examining the relationship between family behavior and GD reported that not only parenting styles but also parental attitudes toward video games can influence the risk of GD (Rosales-Navarro and Torres Pérez, 2025). In other words, parents’ perceptions of the younger generation, their parenting styles, and their attitudes toward gaming can directly or indirectly affect young people’s media use and cultural participation, potentially leading to overdependence and related problems. These findings suggest that issues such as GD are intricately intertwined with the relational and perceptual dynamics between older and younger generations surrounding media use. Therefore, more rigorous empirical investigations are needed to deepen our understanding of these complex intergenerational interactions. While previous studies have provided valuable insights into the clinical and psychosocial dimensions of GD, quantitative investigations examining the sociocultural acceptance of GD and the impact of moral panic remain scarce. In contrast, MP studies to date mostly rely on qualitative analysis. For this reason, it has been difficult to determine whether MP significantly affects negative game attitudes or favorable attitudes toward game regulation. Considering that MP is a factor that prevents sound discussion of gaming disorders and the creation of effective alternatives, basic research to understand the correlation between the two factors is urgently needed.

### 2.4 Current study

The impact of MP may depend on cultural and social tolerance and norms toward a particular target. Differences in perceptions and concerns between generations toward gaming can be found in other countries, but they can be particularly pronounced in East Asian countries (e.g., Korea and China; Jeong et al., 2019). Particularly, in South Korea, where game culture has rapidly spread to the younger generation, the prevalence of hostile perception toward games, i.e., ‘game phobia’, has been pointed out as a major factor preventing healthy discussion of game culture (Kang et al., 2013). For example, studies conducted in South Korea support the fact that there is a difference in perception and generational conflict between the new media generation and the old media generation (Kim and Doh, 2014; Park and Ryu, 2018).

According to one study, in a survey targeting a group of parents of children and adolescents, the parents’ perception toward games was generally negative, and they tend to assume a child’s game addiction to be a greater concern than likely justified by actual clinical symptoms (Choi et al., 2011). Other studies also show that the older the group with less game experience, the more negative gaming is perceived and the simpler the criteria applied for labeling gaming as addictive (Kim et al., 2013). Another study verified that age also affects negative perceptions toward gamers (Park and Ryu, 2018). These findings support the fact that there is a perception gap between generations centered on games in Korean society. In other words, the older the person, the more abstract and threatening the harmfulness of the game, and the negative view of the game player.

In a similar vein, there is also an argument that cultural differences between generations in Korea affect the distorted media perception of the parents. According to the study (Kang et al., 2013), the older generation’s view that gaming is related to socio-cultural problems such as addiction, antisocial behavior, and obesity stems from negative views on leisure time, which has a negative impact on academic, work, and personal performance. In particular, South Korea is a country with very high social pressures on academic performance, including university entrance exams. Consequently, the negative attitudes of the older generation toward digital games are consistent with the general view of a hostile evaluation of leisure time taking away from study. In this regard, one study asserts that the Korean government’s active regulation of the game industry is closely related to the perception of the parent generation regulating their children’s game behavior (Park and Ryu, 2018).

Summarizing the above research, it can be found that the older generation in Korea perceives new media games as unfamiliar objects and reacts sensitively to the threat of games. Therefore, this study investigated the influence of MP by conducting a survey of 2,000 subjects in South Korean. Specifically, this study examined the effects of both responsibility to media threats and negative attitude toward youth on the favor of the decision on the inclusion of GD in ICD-11 as a mental illness (shortly, the degree of agreement to pathologizing gaming disorder) through negative attitudes toward digital games. Following the above arguments, this study tested hypotheses and a research question below:

*H1(a/b)*: (a) Responsibility to media threats and (b) negative attitudes toward youth will increase the degree of agreement to pathologizing gaming disorder.

*H2(a/b)*: (a) Responsibility to media threats and (b) negative attitudes toward youth will increase the degree of negative attitudes toward digital games.

*H3*: Negative attitudes toward digital games will enhance the degree of agreement to gaming disorder.

*RQ1(a/b)*: Negative attitudes towards digital games will mediate the relationship between (a) responsibility to media threats and agreement to pathologizing gaming disorder, and (b) negative attitudes towards youth and agreement to pathologizing gaming disorder.

*RQ2*: How will sex, age, and gaming time affect the degree of negative attitudes toward digital games and the degree of agreement to pathologizing gaming disorder?

## 3 Methods

### 3.1 Participants

This study utilized the data collected by the Korea Creative Content Agency (KOCCA) for a survey on public perceptions of gaming disorder. The data collection received the ethical approval of the Institutional Review Board (IRB) in Konkuk University. A total of 2,000 responses were collected from 15 years old to 60 years old through a specialized research company in Korea for a cognitive survey. Although the optimal determination of sample size in structural equation modeling depends on factors such as the number of latent variables and the complexity of the model, or may require conducting Monte Carlo simulations, it is generally recognized that a sample size of 200 or more is sufficient (Hoelter, 1983; Kyriazos, 2018). Considering the complexity of the model, the present study possesses a sample size that greatly exceeds this commonly accepted empirical rule, and therefore it was judged that there is no problem in conducting the SEM analysis. Only adolescents aged 15 years or older who obtained parental consent were allowed to participate in the study. The purpose of examining various age groups is to confirm and review previous studies that negative perceptions toward new media and the generation gap are significantly related (Kneer et al., 2018; Ferguson and Colwell, 2017). In order to secure representativeness of the sample, a ‘quota sample’ was conducted for gender and age based on resident population registration statistics. A quota sampling method was applied based on age and gender ratios, as media moral panic phenomena are likely to be affected by age differences or negative perceptions toward other generations. The informed consent was obtained from the participants, and all the participants received a small gift certificate in return for participating in the survey.

As a result of the response, 1,016 (50.8%) males and 984 (49.2%) females out of a total of 2000 participants. Also, 136 (6.8%) in their teens, 342 (17.1%) in their 20s, 359 (18%) in their 30s, 423 (21.2%) in their 40s, 434 (21.7%) in their 50s, and 306 (15.3%) in their 60s. appear. And among the 2000 participants, 1,620 (81%) used digital games, and 380 (19%) answered that they did not use the game. The average daily gaming time of the whole sample was 1.59 h (SD = 1.907). At this time, the non-game user was asked to check the game time as ‘0’. As a result of the analysis, a total of 380 respondents reported that they did not play games at all, and no issues related to skewed or zero-inflated distribution of gaming time were identified.

### 3.2 Measures

Responsibility to media threats. Based on the theoretical review, this study organized “responsibility to media threats” into items to confirm protection and responsibility for the younger generation, and concerns and rejection of the negative effects of new media. Since there were few empirical studies on MP and responsibility to media threats, this study produced a total of six measurement items by referring to related previous studies. The scale consists of a Likert 5-point scale (1: strongly disagree – 5: strongly disagree), with “older generations should be actively involved in the problems of children and adolescents,” and “Media that can have a toxic effect on children and young people should be sanctioned,” “Adults have an obligation to protect children and adolescents from potential harm,” Responsible adults should fulfill their duty to protect children and adolescents.” Also included were items such as “It is necessary to regulate harmful media frequently used by children and adolescents through policy,” “It is necessary to actively monitor the media to protect children and adolescents.” All items were presented in Korean.

Negative attitudes toward digital games. Negative attitudes toward digital games were constructed so that the attitude of respondents toward negative dysfunction that games could have on individuals or society could be measured. To measure this, Quandt et al. (2015) and Ferguson and Colwell (2017) used 4 items from the negative attitudes toward digital games scale. The scale consisted of a Likert 5-point scale (1: strongly disagree – 5: strongly agree), “Digital games have a negative impact on society,” and “The addiction effect of digital games on kids and teens are a problem for society,” “The influence of digital games on users’ aggressive behavior is a social issue,” “Digital games may harm the mental health of children and adolescents” were included. The original items were translated into Korean by the researcher, and the translated version was subsequently reviewed and revised by a peer researcher.

Negative attitudes toward youth. Negative attitudes toward youth refer to an individual’s attitude that the qualities of the young generation are worsening compared to the past. For the measurement, three items were used among the negative attitudes toward youth scale made by Ferguson (2015). The items were constructed on a Likert 5-point scale (1: Strongly disagree – 5: Strongly agree), “Youth violence is as high as it has ever been” and “Kids and teens today have more behavioral problems than before.” The scale was also translated into Korean by the researcher, reviewed through peer evaluation, and then administered to the participants.

Agreement to pathologizing gaming disorder. The Agreement to WHO’s decision on GD scale consisted of asking if they agreed with the WHO’s decision on GD and whether GD should be officially recognized as a disease. The scale consisted of 3 items (e.g., “Do you agree with the WHO registration of GD as a disease?” and “Do you think that Korea should recognize GD as a disease like the WHO decision?”). It was also measured on a Likert 5-point scale (1: Strongly disagree – 5: Strongly agree).

## 4 Data analysis

All analyses were conducted using AMOS 22.0 and SPSS 22.0. A two-step approach (Anderson and Gerbing, 1988) was adopted to test the proposed structural equation model (SEM). In the first step, a measurement model was examined to assess the reliability and validity of each latent construct, and in the second step, a structural model was estimated to verify the hypothesized relationships among variables. To assess the measurement model, confirmatory factor analysis was conducted using the maximum likelihood estimation method. The results of the measurement model test are presented in Table 1. The measurement model demonstrated an acceptable fit to the data (*χ*^2^ = 1276.14, df = 98, *χ*^2^/df = 13.02, CFI = 0.952, TLI = 0.941, NFI = 0.948, RMSEA = 0.078, 90% CI [0.074, 0.081]). Although the chi-square statistic was significant, this index is sensitive to sample size. The other fit indices indicated that the measurement model showed an overall satisfactory fit, supporting the adequacy of the measurement structure for subsequent structural equation modeling. Tests included Cronbach’s alpha, composite reliability (CR), and average variance extracted (AVE), and discriminant validity of the constructs. As a result of the analysis, the Cronbach’s alpha value was found to be greater than 0.7, indicating that it was valid (Nunnally and Bernstein, 1994). Along with this, CR and AVE scores were also found to be suitable for use in testing (0.8 for CR and 0.5 for AVE; Chin, 1998). Based on the Fornell–Larcker criterion, discriminant validity was established. The square roots of the average variance extracted (AVE) for all constructs (ranging from 0.797 to 0.923) exceeded their respective inter-construct correlations (ranging from 0.203 to 0.578), indicating satisfactory discriminant validity among the latent variables (Fornell and Larcker, 1981). The discriminant validity results for the constructs are shown in Table 2.

**Table 1:** Reliability and correlation analysis.

Variables	N	M	SD	α	1	2	3	4	5	6	7
Sex (0 = male, 1 = female)	–	0.49	0.50	–	**–**						
Age	–	42.71	14.54	–	0.026	**–**					
Gaming time	–	1.59	1.907	**–**	−0.110**	−0.349**	**–**				
Negative attitudes toward digital games	4	3.5535	0.8603	0.875	0.270**	0.367**	−0.237**	**–**			
Negative attitudes youth	3	3.9442	0.8318	0.837	0.200**	0.336**	−0.115**	0.506**	**–**		
Agreement to pathologizing GD	3	3.5250	0.9826	0.947	0.251**	0.322**	−0.223**	0.648**	0.417**	**–**	
Responsibility to media threats	6	4.019	0.7356	0.912	0.228**	0.375**	−0.224**	0.680**	0.540**	0.544**	**–**

**Table 2:** Correlations and discriminant validity analysis.

Variables	1	2	3	4	AVE	CR
Responsibility to media threats	–				0.700	0.9333
Negative attitudes youth	0.3422	–			0.688	0.8666
Negative attitudes toward digital games	0.5776	0.3364	–		0.635	0.8741
Agreement to pathologizing GD	0.3271	0.2025	0.4984	–	0.851	0.9452

Based on the conceptual model developed from the research hypotheses, a structural equation modeling analysis was conducted, and a bootstrapping procedure was employed to examine the mediation effects. The analysis results showed that the model fit values were found to be at valid levels (*χ*^2^ = 1885.727, df = 125, *χ*^2^/df = 15.086, IFI = 0.931, TLI = 0.906, CFI = 0.931, NFI = 0.927 and RMSEA = 0.084, 90% CI [0.081, 0.087]).

## 5 Results

**Figure 1:**
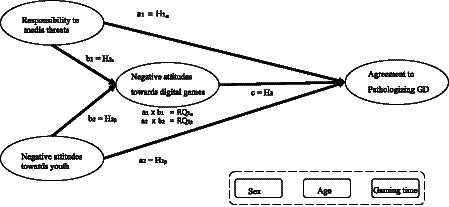
A conceptual model.

As a result of the SEM analysis, responsibility to media threats was significantly and positively associated with negative attitudes toward digital games (*β* = 0.711, *p* < 0.001; see Figure 1 and Table 3). Responsibility to media threats was also significantly associated with agreement that the GD concept is valid (*β* = 0.154, *p* < 0.001). Accordingly, both H1a and H2a were supported. In addition, negative attitudes toward youth were significantly correlated with both negative attitudes toward digital games and agreement that the GD concept is valid, respectively (*β* = 0.160 and 0.081, *p* < 0.001). These findings indicate that both H1a and H2a were confirmed. Likewise, negative attitudes toward digital games were positively related to agreement that the GD concept is valid (*β* = 0.467, *p* < 0.001). Therefore, H3 was accepted. Taken together, these associations indicate that individuals perceiving higher responsibility for media threats tend to report stronger negative attitudes toward games and greater endorsement of the gaming disorder concept, although causal relationships cannot be inferred from the present cross-sectional design.

**Table 3:** Results of SEM test.

Hypothesis	*B*	*β*	S.E.	C.R.
Sex → Negative attitudes toward digital games	0.168	0.109	0.025	6.634***
Age → Negative attitudes toward digital games	0.004	0.071	0.001	4.103***
Gaming time → Negative attitudes toward digital games	−0.21	−0.053	0.007	−3.025**
Sex → Agreement to pathologizing GD	0.136	0.08	0.031	4.333***
Age → Agreement to pathologizing GD	0.004	0.068	0.001	3.562***
Gaming time → Agreement to pathologizing GD	−0.024	−0.052	0.009	−2.72**
Responsibility to media threats → Negative attitudes toward digital games	1.249	0.711	0.061	20.448***
Responsibility to media threats → Agreement to pathologizing GD	0.302	0.154	0.067	4.514***
Negative attitudes toward youth → Negative attitudes toward digital games	0.229	0.16	0.025	8.999***
Negative attitudes toward youth → Agreement to pathologizing GD	0.128	0.081	0.032	4.056***
Negative attitudes toward digital games → Agreement to pathologizing GD	0.52	0.467	0.043	12.07***

*p < 0.05, **p < 0.01, ***p < 0.001.

**Figure 2:**
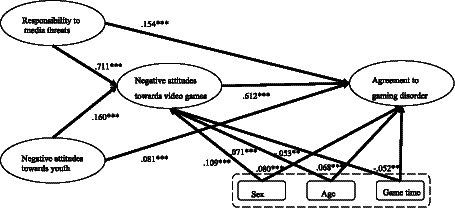
Structural equation model.

Among all the examined associations, the strongest relationship was observed between responsibility to media threats and negative attitudes toward digital games (*β* = 0.711), while demographic variables such as sex (*β* = 0.109) and age (*β* = 0.071) showed small but statistically significant associations. The average daily gaming time exhibited a small negative association with both negative attitudes toward digital games and agreement with the GD concept (*β* = −0.053 and −0.052, *p* < 0.01). Overall, these results highlight that media-related perceptions are more strongly associated with attitudinal variables than demographic characteristics (see Figure 2).

Regarding the strength of associations, the standardized coefficients ranged from *β* = −0.053 to *β* = 0.711, reflecting small to large magnitudes of association (Cohen, 2002). The strongest relationship was observed between responsibility to media threats and negative attitudes toward digital games (*β* = 0.711), representing a large and practically meaningful association, while demographic variables such as sex (*β* = 0.109) and age (*β* = 0.071) showed small but statistically significant associations. The average daily gaming time exhibited a small negative association with both negative attitudes toward digital games and agreement with the GD concept (*β* = −0.053 and −0.052, *p* < 0.01). Overall, these findings describe relational patterns among variables rather than causal effects, highlighting that media-related perceptions are more strongly associated with attitudinal variables than demographic characteristics.

The analysis revealed that responsibility to media threats had a significant effect on negative attitudes toward digital games, and likewise, Negative attitudes toward digital games significantly influenced agreement to pathologizing GD. Accordingly, the mediating effect of negative attitudes toward digital games was examined using confidence intervals (CIs) obtained through a bootstrapping procedure to evaluate the statistical significance of each indirect effect. The bootstrap analysis was conducted with 5,000 resamples.

The results of the bootstrapped mediation analysis indicated that the indirect effect of responsibility to media threats on agreement to pathologizing GD through Negative attitudes toward digital games was statistically significant (*β* = 0.332, *SE* = 0.029, 95% CI [0.277, 0.394]). This finding suggests that higher levels of responsibility to media threats strengthen negative attitudes toward digital games, which in turn increase agreement with pathologizing game disorder. In addition, the indirect effect of Negative attitudes toward youth on agreement to pathologizing GD through negative attitudes toward digital games was also significant but relatively weaker (*β* = 0.075, *SE* = 0.015, 95% CI [0.048, 0.108]).

## 6 Discussion

### 6.1 Key findings

This study reviewed the major theories and key factors related to MP and was conducted to empirically examine whether the core components that constitute moral panic influence negative attitudes toward games and opinions on game regulation policies. The impact and dynamics of MP are likely to be further intensified within the distinctive socio-cultural context of Korea. In a society where social pressure for academic achievement is exceptionally high, the older generation has historically viewed gaming as a harmful practice that distracts from scholastic achievement or as a wasteful leisure activity. Consequently, in cultural environments such as Korea—where vigilance toward new media is strong and concerns about academic performance are heightened—parents and educators may be more susceptible to moral panic and more receptive to newly introduced regulations on game use (Jeong et al., 2019; Kang et al., 2013; Kim and Doh, 2014; Lee and Lason, 2009; Park and Ryu, 2018). Yet, empirical studies directly investigating these processes have been limited. Given the rapid cycles of new-media replacement driven by technological advances such as digital games, the metaverse, and AI, the need for such research has become increasingly urgent.

According to previous studies on MP, negative attitudes toward new media tend to arise from a sense of responsibility for socio-cultural values (i.e., youth protection) and negative perceptions of youth (Goode and Ben-Yehuda, 2009; Pascoe, 2011). Building on this theoretical background, the present study examined how responsibility to media threats and negative attitudes toward youth influence negative attitudes toward digital games and agreement with the pathologization of GD. The results indicated that both negative attitudes toward youth and responsibility to media threats were positively associated with negative attitudes toward digital games and support for regulatory policies. Notably, responsibility to media threats showed a stronger association with negative attitudes toward digital games than negative attitudes toward youth. A similar tendency was found for agreement with the pathologization of gaming disorder, as responsibility to media threats demonstrated a larger coefficient. The finding that responsibility to media threats acts as a stronger predictor suggests that regulatory support may be driven less by intergenerational prejudice and more by an overprotective sense of moral duty to safeguard youth from perceived risks of new media. Presumably, this result may be because the sense of protection toward the youth motivates the decision of attitude toward new media more than the negative prejudice against the younger generation. Perhaps prejudice against the younger generation can lead to an underestimation of their culture, but it can be difficult to directly elicit an attitude or regulatory support for the harmfulness of the media. For example, a negative attitude toward youth can lead to various reactions, such as distorting the influence of subcultures or ignoring them altogether.

As a result of examining the relationship between demographic variables and negative gaming attitudes, both gender and age showed a positive relationship with negative gaming attitudes, but it was confirmed that game time had a negative relationship. More specifically, women, older age, and less game time were positively related to negative game attitudes. And these results were also found in the relationship with agreement to gaming disorder. Since the main users of digital games are young men, these results are consistent with the theoretical approach that the more unfamiliar with new media, the more overestimated the risks of games.

In addition, negative attitudes toward digital games were found to partially mediate the effects of responsibility to media threats and negative attitudes toward youth on agreement with the pathologization of GD. This indicates that socio-cultural anxieties—such as prejudice against younger generations and concerns about harmful media—influence regulatory support by shaping hostility toward new media. In other words, cultural atrophy within digital game discourse and public acceptance of premature regulatory measures may reflect, at least in part, the entrenched negative societal perceptions of digital games. These perceptions may have been amplified and reproduced through the older generation’s concerns about harmful media and the broader intergenerational divide.

### 6.2 Theoretical implications

This study makes several significant theoretical contributions to the literature on MP. First, our findings empirically validate the MP framework, confirming that negative attitudes toward new media arise from a sense of responsibility for socio-cultural values and negative attitudes toward youth. Consistent with Ferguson’s (2015) assertion that preconceived notions toward youth can negatively prejudice perceptions of their culture, our results show that the older generation’s protective anxiety forms a “moral barricade,” reinforcing excessive fear of new media. Crucially, this study refines the MP model by distinguishing between “negative attitudes toward youth” and “protective moral responsibility.” While both factors are significant, our analysis reveals that responsibility to media threats is a stronger and more direct predictor of regulatory support than negative attitudes toward youth.

Theoretically, this suggests that moral panic is driven less by simple intergenerational animosity and more by a perceived moral obligation to protect vulnerable youth. As previously discussed, prejudice may lead to passive reactions such as ignoring subcultures, whereas a sense of duty to “lead” the younger generation mobilizes more active regulatory responses. This distinction is important for future MP models because it underscores that the motivation behind pathologization policies stems primarily from a desire to protect, rather than from mere dislike. In addition, the finding that negative attitudes toward digital games partially mediate these relationships sheds light on the cognitive mechanism of MP. The results indicate that abstract socio-cultural anxieties do not directly translate into policy support; instead, they influence public judgments through the consolidation of negative media frames, such as hostile attitudes toward games. This mediating pathway demonstrates how broad societal fears become transformed into targeted antagonism toward a specific medium, which subsequently reinforces the rationale behind the Gaming Disorder diagnosis.

Finally, the results regarding demographic variables support the theoretical premise that distance from new media correlates with heightened risk perception. The finding that women, older adults, and non-gamers hold more negative attitudes aligns with the view that unfamiliarity with a medium breeds overestimation of its harm. This reinforces the argument that the generation gap in MP is fundamentally a experience gap, where the lack of direct engagement with the medium amplifies fear and reliance on regulatory control.

### 6.3 Limitations

Despite its contributions, this study has several limitations that should be acknowledged. First, the cross-sectional design prevents strong causal inferences regarding the relationships among socio-cultural concerns, negative game attitudes, and support for the pathologization of Gaming Disorder. Although the proposed model is theoretically grounded in the MP framework, longitudinal or experimental designs would be necessary to determine the temporal ordering and causal mechanisms underlying these effects. Second, while the study incorporated key sociological constructs—responsibility to media threats and negative attitudes toward youth—it was not able to include a wider range of MP-related variables such as media exposure patterns, political ideology, social values. These factors may interact with MP in shaping regulatory attitudes and should be examined in future research to build a more comprehensive model in digital media contexts. Third, the findings reflect the distinct socio-cultural characteristics of South Korea. Although this cultural specificity provides valuable insight into how MP discourse manifests in East Asian contexts, it may limit generalizability to other societies. Comparative cross-cultural studies would be beneficial to determine whether similar mechanisms operate in Western or other non-Korean contexts. Finally, the study relied on self-reported data, which may be affected by social desirability. Future research may integrate behavioral measures, media usage logs, or qualitative interviews to triangulate findings.

### 6.4 Conclusion

This study advances the theoretical understanding of MP by empirically demonstrating that its core components play a significant role in shaping negative perceptions of digital games and support for the pathologization of gaming disorder. The findings highlight the distinct mechanisms through which socio-cultural concerns translate into regulatory attitudes, showing that protective moral responsibility functions as a more powerful predictor of regulatory endorsement than generational prejudice alone. Additionally, the mediating role of negative attitudes toward digital games clarifies the cognitive pathway through which diffuse socio-cultural anxieties crystallize into concrete policy preferences.

Practically, these findings suggest that effective policy approaches should move beyond preemptive regulation and instead focus on reducing the generational perception gap, particularly through digital literacy initiatives for parents and educators. Such efforts may help alleviate unfounded fears that drive negative attitudes toward emerging media. Taken together, the results indicate that contemporary debates surrounding digital games cannot be fully understood without considering the broader socio-cultural anxieties embedded in intergenerational relations and media-related concerns. As digital media environments continue to evolve rapidly, future research should examine how these dynamics unfold across diverse cultural contexts and among populations with varying levels of media familiarity.

## Data Availability

The raw data supporting the conclusions of this article will be made available by the authors, without undue reservation.
